# Postural deformities in Parkinson’s disease –Mutual relationships among neck flexion, fore-bent, knee-bent and lateral-bent angles and correlations with clinical predictors

**DOI:** 10.1186/s40734-016-0029-8

**Published:** 2016-01-29

**Authors:** Fumihito Yoshii, Yusuke Moriya, Tomohide Ohnuki, Masafuchi Ryo, Wakoh Takahashi

**Affiliations:** Department of Neurology, Tokai University Oiso Hospital, 21-1 Gakkyou, Oiso, Naka-gun, Kanagawa 259-0198 Japan

**Keywords:** Parkinson’s disease, Postural deformity, Hoehn and Yahr (H&Y) stage, Levodopa equivalent dose, Mutual correlations, Balance

## Abstract

**Background:**

Various postural deformities appear during progression of Parkinson’s disease (PD), but the underlying pathophysiology of these deformities is not well understood. The angle abnormalities seen in individual patients may not be due to distinct causes, but rather they may have occurred in an interrelated manner to maintain a balanced posture.

**Methods:**

We measured the neck flexion (NF), fore-bent (FB), knee-bent (KB) and lateral-bent (LB) angles in 120 PD patients, and examined their mutual relationships, and correlations with clinical predictors such as sex, age, disease duration, Hoehn and Yahr (H&Y) stage, medication dose (levodopa equivalent dose, LED; total dose of dopamine agonists, DDA). The relationship between the side of the initial symptoms and the direction of LB angle was also investigated.

**Results:**

Our main findings were: (1) Significant relationships between NF and KB, NF and LB, FB and KB, KB and LB were observed. (2) NF angle was larger in males than in females, but FB, KB and LB angles showed no significant difference between the sexes. (3) FB and KB angles became larger with advancing age. (4) NF and FB angles were associated with disease duration. (5) NF, FB, KB and LB angles all increased significantly with increase of H&Y stage. (6) FB angle was significantly associated with LED, but DDA did not show a significant relationship with any of the measured angles. (7) Direction of LB angle was not associated with the side of initial symptoms.

**Conclusions:**

Postural abnormalities are interrelated, possibly to maintain a balanced posture.

## Background

Postural deformities are frequent and disabling complications of Parkinson’s disease (PD) and interfere with daily living activities, often leading to falls. These deformities include antecollis (dropped head), camptocormia (stooped or bent posture; marked bending of the thoraco-lumbar spine), and lateral flexion (Pisa syndrome) [1]. In addition, knee flexion is often observed.

Although various types of postural deformities have been reported in patients with PD, the underlying pathophysiology of these deformities is not well understood and a number of different causes have been proposed. However, the angle abnormalities seen in individual patients may not be due to distinct causes, but rather they may have occurred in an interrelated manner to maintain a balanced posture. For example, combinations of deformities, such as dropped head and lateral flexion, are often observed simultaneously. Here, we measured the neck flexion (NF), fore-bent (FB), knee-bent (KB) and lateral-bent (LB) angles in 120 PD patients, in order to establish their mutual relationships. We also examined the relationships of these angles to several clinical predictors, such as sex, age, disease duration, disease severity, and dose of medicine, in order to clarify possible contributory pathophysiological mechanisms.

## Methods

### Subjects

We enrolled 120 patients (age: 41–88 years old, average: 67 ± 10 years old) with PD who were able to maintain a standing position without assistance (66 males, age 41–87 years, average 66 ± 11 years; 54 females, age 43–88 years, average: 68 ± 8 years). A diagnosis of PD was made according to the United Kingdom Parkinson’s Disease Society Brain Bank Clinical Diagnostic Criteria. Disease duration was 0–21 years (8.2 ± 5.1 years). Hoehn & Yahr’s severity scale “on stage” (H&Y stage) was 2.7 ± 0.8. Information about the laterality of the initial symptom at onset was extracted from clinical records. Brain magnetic resonance imaging (MRI) was performed in all individuals to rule out multiple system atrophy (MSA) [2] or progressive supranuclear palsy (PSP) [3], which frequently show similar postural deformities. Comorbid orthopedic spinal lesions (compression fracture of the vertebrae, disc hernia, or spondylolisthesis of the vertebrae) were diagnosed on anteroposterior and lateral views of cervical, thoracic and lumbar spinal X-ray pictures and five patients with prominent spinal bone disorders were excluded prior to enrollment into the study. Patients who had undergone deep brain stimulation were also excluded, because DBS is used for amelioration of postural deformities in PD [4], and we wished to focus on the intrinsic postural characteristics of PD.

The study was approved by Tokai University Review Board, and all eligible individuals were informed of the purpose and methods of the study. All of them provided written informed consent.

### Measurement of NF, FB, KB and LB angles

**Fig. 1:**
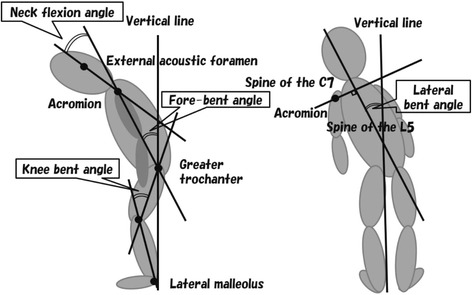
Schematic representation of body angles. In lateral view photographs (left), NF (neck flexion) angle was defined as the angle between two intersecting lines: a line connecting the external acoustic foramen and the acromion, and another line connecting the acromion and the greater trochanter. Similarly, FB (fore-bent) angle was defined as the angle between a vertical line and the line connecting the acromion with the greater trochanter. KB (knee-bent) angle was defined as the angle between the line connecting the greater trochanter and knee, and the line connecting knee and lateral malleolus. In back view photographs (right), LB (lateral-bent) angle was defined as the angle between a vertical line and the line connecting the posterior process of the seventh cervical vertebra with that of the fifth lumbar vertebra

In the present study, four body angles, i.e., neck flexion (NF), fore-bent (FB), knee-bent (KB) and lateral-bent (LB) angles, were defined with reference to the report of Oeda et al. [5]. NF, FB, KB and LB angles were measured on photographs of the lateral and back views of the patients in an upright position (in an “on” period if the patients had motor fluctuations). In lateral view photographs, the angle between two intersecting lines — the line connecting the external acoustic foramen and the acromion, and the line connecting the acromion and the greater trochanter—was defined as the NF angle. Similarly, FB angle was defined as the angle between the line connecting the acromion and the greater trochanter, and a vertical line. KB angle was defined as the angle between the line connecting the greater trochanter and knee, and the line connecting knee and lateral malleolus. In back view photographs, the angle between the line connecting the posterior process of the seventh cervical vertebra and that of the fifth lumbar vertebra, and a vertical line was defined as the LB angle (Fig. 1). NF and FB angles toward flexion and extension were expressed as positive and negative values, respectively, and LB angle was shown as an absolute value, because bending toward the left or right sides appears to occur by chance.

### Data collection

Clinical data including sex, age, disease duration, H&Y stage, medication dose (levodopa equivalent dose, LED; total dose of dopamine agonists, DDA) and side of initial (prominent) parkinsonian symptoms (right or left) were collected as potential predictors of abnormal posture.

Daily doses of levodopa, dopamine agonists, selegiline entacapone and amantadine at study entry were also recorded. The LED was calculated according to the formula: LED (mg) = levodopa (mg) + pramipexole (mg) × 100 + ropinirole (mg) × 25 + pergolide (μg) × 0.1 + cabergoline (mg) × 67 + bromocriptine (mg) × 10. For patients treated with selegiline, their levodopa dose was multiplied by 1.30, and for those taking entacapone, by 1.25. The DDA was calculated according to the formula: pramipexole (mg) × 100 + ropinirole (mg) × 25 + pergolide (μg) × 0.1 + cabergoline (mg) × 67 + bromocriptine (mg) × 10.

### Statistical analysis

Differences of NF, FB, KB and LB angles between male and female PD patients were compared using the Mann–Whitney *U* test. Correlations of the angles with age, disease duration, H&Y stage (on) and medication dose (LED, DDA) were examined using Spearman’s rank correlation coefficients. The relationship between onset side and LB was examined with the Mann–Whitney *U* test. Mutual relationships of NF, FB, KB and LB angles were investigated using Spearman’s rank correlation coefficients. We confirmed that the population was not normally distributed by use of the Kolmogorov-Smimov test before running non-parametric tests. A value of *p* < 0.05 was considered statistically significant. Statistical analyses were performed using the SPSS-21 software package (IBM).

## Results

### Body angles in PD patients

**Fig. 2:**
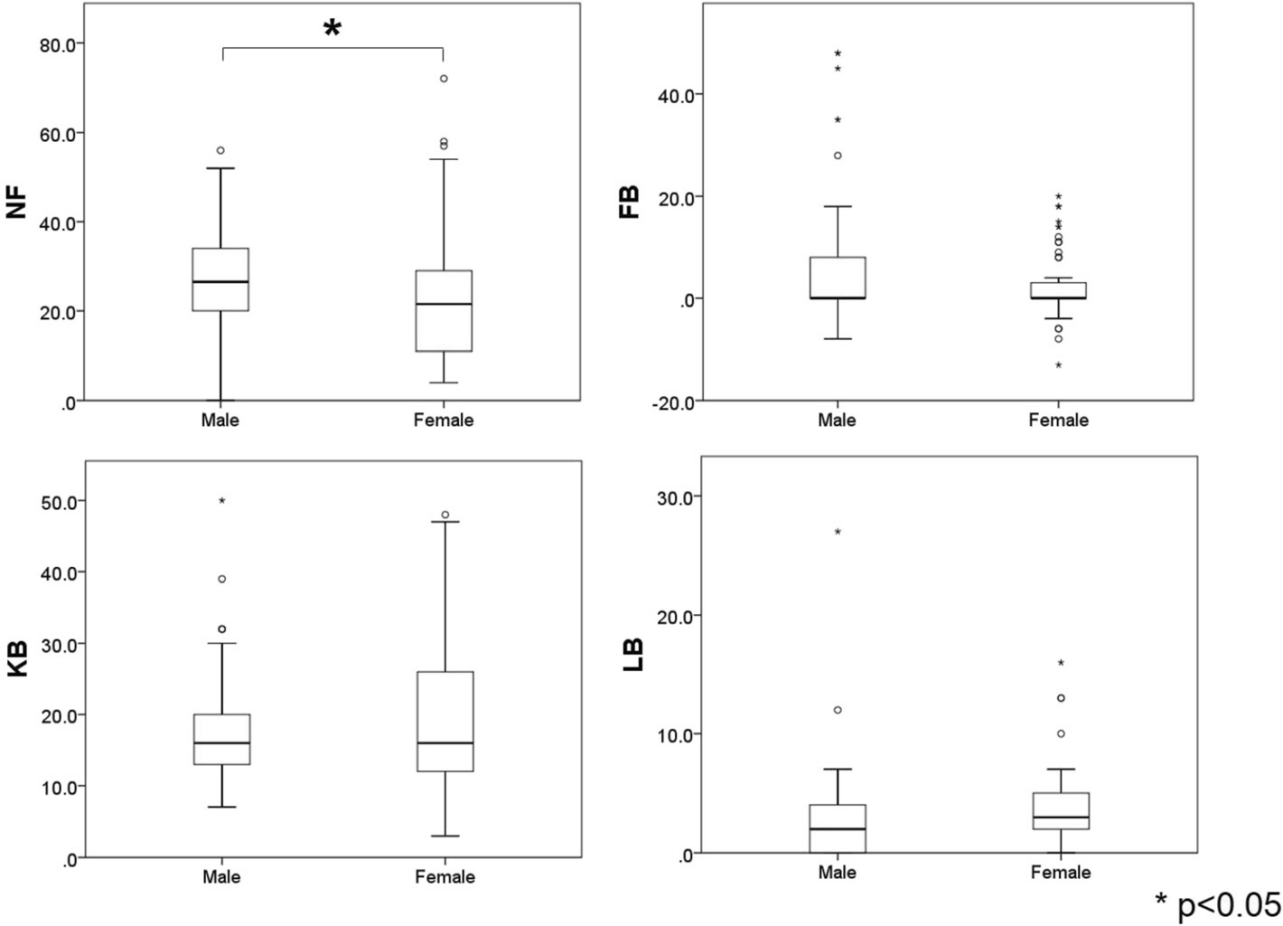
NF, FB, KB and LB angles in male and female PD patients. NF angle was significantly larger in males than in females (*P* < 0.05), but FB, KB and LB angles showed no significant difference between males and females

NF, FB, KB and LB angles in male and female patients with PD are shown in Fig. 2. We found that age (male: 66 ± 11 years old, female: 68 ± 8 years old), disease duration (male: 7.8 ± 4.7 years, female: 8.6 ± 5.4 years), H&Y stage (male: 2.8 ± 0.8, female: 2.7 ± 0.7) and LED (male: 501 ± 253 mg, female: 436 ± 233 mg) were not significantly different between genders. NF angle for males (27.6 ± 11.8) was significantly greater than in females (22.9 ± 14.9) (*p* = 0.01). However, other angles were not significantly different between males and females (FB: 5.4 ± 11.6 and 2.2 ± 6.7, KB: 17.7 ± 7.7 and 19.6 ± 10.2, LB: 2.7 ± 3.9 and 3.5 ± 3.4, respectively).

### Effects of age, disease duration, H&Y stage and LED

Correlations between NF, FB, KB, LB angles and clinical predictors (age, disease duration, H&Y stage and LED) are shown in Table 1.

**Table 1:** Correlation coefficients between NF, FB, KB, LB angles and clinical predictors

	Age	Disease Duration	H&Y stage	LED
NF	0.175	0.197 ^*^	0.350 ^***^	0.135
FB	0.246 ^**^	0.196 ^*^	0.492 ^***^	0.243 ^**^
KB	0.359 ^***^	0.098	0.381 ^***^	0.111
LB	0.162	0.103	0.213 ^*^	0.173

FB and KB angles increased significantly with advancing age. NF and FB angles were significantly associated with disease duration. All the measured angles increased significantly with advancing H&Y stage. There was a significant association between LED and FB, but not the other angles

^*^ *P* < 0.05, ^**^ *P* < 0.01, ^***^ *P* < 0.001

FB (*r* = 0.246, *p* < 0.01) and KB (*r* = 0.359, *p* < 0.001) angles increased significantly with advancing age. Further, NF (*r* = 0.197, *p* < 0.05) and FB (*r* = 0.196, *p* < 0.05) angles were significantly associated with disease duration. All the measured angles increased significantly with advancing H&Y stage: NF (*r* = 0.350, *p* < 0.001), FB (*r* = 0.492, *p* < 0.001), KB (*r* = 0.381, *p* < 0.001) and LB (*r* = 0.213, *p* < 0.05). Finally, there was a significant association between LED and FB (*r* = 0.243, *p* < 0.01), but not the other angles. DDA showed no significant relationship with any of the measured angles. Mean values of LED and DDA were 472 ± 248 mg/day and 123 ± 120 mg/day, respectively.

Multiple regression analysis was performed to assess the relationships among these four predictors as explanatory variables and each angle as a categorical response variable. H&Y stage was the most explanatory variable for NF (partial regression coefficient (B) = 6.578, *p* < 0.001), FB (B = 5.976, *p* < 0.001) and KB (B = 5.614, *p* < 0.001), while LED was the most explanatory variable for LB (B = 0.004, *p* = 0.002).

### Laterality of initial symptom and LB angle

**Fig. 3:**
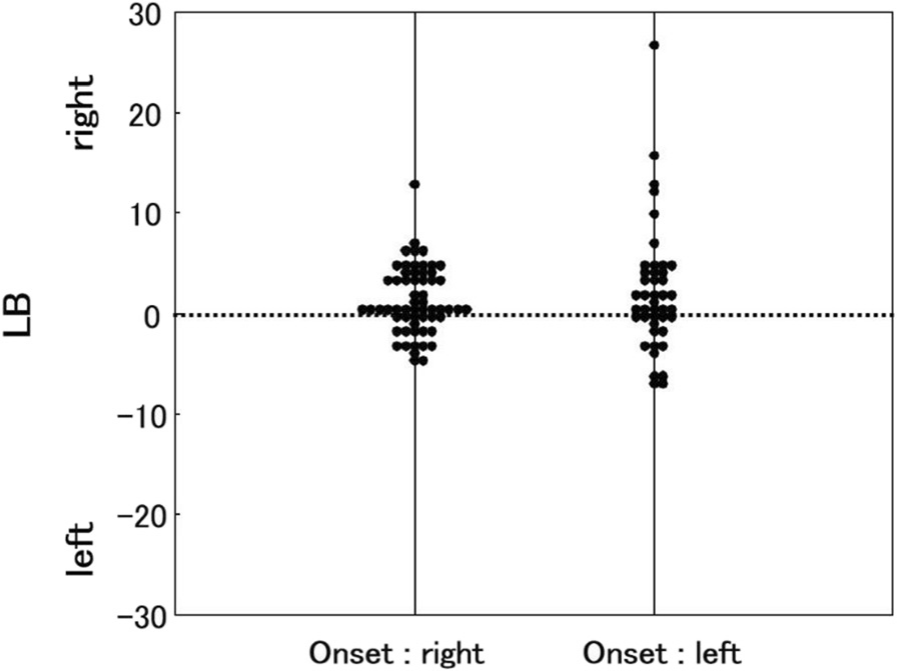
The relationship between laterality of the initial symptom and LB angle. Bending side (direction of LB angle) was not associated with the side of the initial symptom

The relationship between laterality of the initial symptom and LB angle was examined. For 59 patients, the initial symptom was on the right side and for 43 patients, it was on the left (almost symmetrical 15, unknown 3). Bending side (direction of LB angle) was not associated with the side of the initial symptom (Fig. 3).

### Mutual relationships among measured angles

Table 2 shows the relationships among the measured angles. NF and KB (*r* = 0.275, *p* < 0.01), NF and LB (*r* = 0.250, *p* < 0.01), FB and KB (*r* = 0.335, *p* < 0.001), KB and LB (*r* = 0.225, *p* < 0.05) were significantly positively correlated.

**Table 2:** Mutual correlation coefficients among the measured angles (All)

	FB	KB	LB
NF	0.166	0.275 ^**^	0.250 ^**^
FB		0.335 ^***^	0.141
KB			0.225 ^*^

NF and KB, NF and LB, FB and KB, KB and LB were significantly positively correlated. FB (camptocormia) showed a particularly strong correlation with KB (bending of the knee)

^*^ *P* < 0.05, ^**^ *P* < 0.01, ^***^ *P* < 0.001

**Table 3:** Mutual correlation coefficients among the measured angles (By age)

<70 years old (*n* = 57)			
	FB	KB	LB
NF	0.111	0.241	0.199
FB		0.234	0.070
KB			0.163
≧70 years old (*n* = 63)			
	FB	KB	LB
NF	0.166	0.245	0.269 ^*^
FB		0.375 ^**^	0.137
KB			0.190

Significant correlations were found only in the older group, between NF and LB, and between FB and KB

^*^ *P* < 0.05, ^**^ *P* < 0.01

**Table 4:** Mutual correlation coefficients among the measured angles (By gender)

Male (*n* = 66)			
	FB	KB	LB
NF	0.271 ^*^	0.228	0.305 ^*^
FB		0.402 ^***^	0.222
KB			0.078
Female (*n* = 54)			
	FB	KB	LB
NF	−0.011	0.359 ^**^	0.286 ^*^
FB		0.295 ^*^	0.095
KB			0.381 ^**^

Significant correlations were found between NF and LB and between FB and KB in both sexes

^*^ *P* < 0.05, ^**^ *P* < 0.01, ^***^ *P* < 0.001

We also examined the relationships among the measured angles separately by age or gender, and the results are shown in Tables 3 and 4. When subjects were divided into a younger group (age less than 70 years old; *n* = 57) and an older group (age over 70 years old; *n* = 63), significant correlations were found only in the older group, between NF and LB (*r* = 0.269, *p* < 0.05) and between FB and KB (*r* = 0.375, *r* < 0.01). As to gender, different trends were found in men (*n* = 66) and women (*n* = 54), but significant correlations were seen between NF and LB and between FB and KB in both sexes.

## Discussion

Postural deformities are frequent complications in PD: 5–6 % for dropped head (antecollis), 3–17.6 % for camptocormia and 8.5–60 % for lateral flexion (Pisa syndrome) [1]. However, frequency of knee flexion has not been reported. In general, epidemiological studies suggest that the prevalence of antecollis or camptocormia might be higher in Asian patients. As to the pathogenesis of these deformities, axial dystonia, muscle rigidity and myopathy have been suggested for camptocormia, and dopaminergic drugs may be associated with dropped head. However, the precise mechanisms have not been elucidated, although myopathy, skeletal and soft tissue changes have been proposed as peripheral processes. Furthermore, it is not clear whether each postural abnormality occurs independently, or whether the abnormalities occur in association at the same time.

We found that the NF angle was significantly larger in males than in females. Although Kashihara et al. found no gender effect on neck flexion (dropped head) [6], the NF angle of males has been reported to be greater than that of females even in normal healthy individuals [5]. In general, the development of PD symptoms in women may be delayed by higher physiological levels of striatal dopamine, possibly due to the activity of estrogens, as indicated by a SPECT imaging study using ^123^I-FR-CIT tracer [7]. Milder motor deterioration or striatal degeneration suggests a more benign phenotype in women, and may be related to the gender difference of NF. We did not check MMSE (Mini-Mental State Examination) score, but another possibility is that men have more cognitive impairment [7], which also influences postural deformities [8]. On the other hand, no consistent trends concerning gender difference of camptocormia were found in previous studies [5, 6, 9, 10], as well as our study, suggesting that some other etiology, such as dystonia, rigidity or comorbid orthopedic spinal lesions, might be implicated in camptocormia.

Previous studies have identified many clinical parameters such as age [8, 10], disease duration [8], LED [10], H&Y stage [8, 10], UPDRS (Unified Parkinson’s Disease Rating Scale)-3 [5, 8, 10], MMSE scores [8], autonomic dysfunction [10], and history of vertebral surgery [5, 8] as risk factors for postural deformities. Most reports show a positive association between camptocormia and aging or disease severity. Tiple et al. [8] studied 275 consecutive PD patients and found that disease severity assessed by H&Y staging score or UPDRS part 3 score, levodopa dose and dementia (DSM-4) were associated with camptocormia. Kashihara et al. [6] reported that advancing age and disease severity may be the major risks for developing postural disorders. Seki et al. [10] showed that camptocormia had significant associations with aging, more severe motor symptoms, higher L-dopa daily dose and autonomic symptoms (severe constipation, urinary incontinence). In the study by Oeda [5], NF angle was associated only with H&Y stage ≥3. Our study revealed significant relationships of age with FB and KB angles, disease duration with NF and FB angles, H&Y stage with all of the measured angles, and LED with FB angle. Multiple regression analysis showed that H&Y staging was the most explanatory variable for NF, FB and KB angles, whereas LED was the most explanatory variable for LB angle.

Recent reports have drawn attention to a possible role of medication-induced changes in posture, particularly for dropped head. In particular, dopamine agonists can induce or aggravate abnormal posture [11, 12]. In our study, multiple regression analysis showed that LED had the greatest effect on LB angle, though DDA was not related to any of the measured angles. Although the dose of the drug is likely to parallel the severity of disease, we believe that there may be a need to consider the LED effect on LB. However, most cases of drug-induced postural changes have been reported from Japan, and this might suggest the involvement of a genetic difference in the expression of drug-metabolizing enzymes and/or transporters. In our study, we excluded patients who were considered to show postural changes potentially related to dopamine agonist treatment.

There has been debate about whether patients with lateral trunk flexion lean towards or away from the side with more prominent parkinsonian symptoms. Most investigators have found that patients tend to lean away from the most affected side. Although the number of subjects in the present study may be insufficient to allow a definitive conclusion, we found no association of LB angle with onset side (prominent parkinsonian symptom side), suggesting that lateral shift of the body in PD is not related to left-right difference of dopamine in the brain. The tilt to the side is thought not to be due to extrapyramidal symptoms such as axial rigidity or dystonia, but is believed to reflect impaired perception of the vertical position [13].

Although angle changes were associated with aging, increased disease severity, etc., it should be considered that they may occur in a compensatory manner in order to maintain postural stability. For example, when camptocormia becomes severe, the neck is flexed less because of compensatory hyperextension to obtain a normal visual field. In the present study, significant correlations were observed between NF and KB, NF and LB, FB and KB, and KB and LB. FB (camptocormia) showed a particularly strong correlation with KB (bending of the knee), suggesting that knee bending may serve to counteract a forward-inclined posture so as to prevent falling. We found that the correlations of the angles varied with age and gender. Significant correlations between NF and LB, and between FB and KB were seen only in the older group (Table 3). These results are consistent with the finding that significant aging-related changes of FB and KB angles occur in PD (Table 1). Interestingly, we found correlations between NF and LB and between FB and KB in both men and women, but other significant correlation found in women were not observed in men (Table 4). The reason for this is not clear, but may be related in part to the gender difference of aging-related motor deterioration [14].

Postural control is a complex system involving integration of sensory information (vestibular, visual and proprioceptive). Proprioception provides highly accurate information that helps to hold the body vertically in healthy people. Many studies have confirmed that proprioceptive function is abnormal in PD [15]. Proprioceptive defects affect axial motor or postural control in the sagittal and coronal planes, which makes turning movements difficult and may eventually lead to a fall or injury.

Abnormal postures often interfere with the daily life activities of PD patients and have many negative consequences. NF may cause difficulty in swallowing, excessive drooling or restricted vision. FB can cause of anorexia through pressure on the stomach or a sensation of tightening in the abdomen. Also, patients might complain of shortness of breath due to restricted lung capacity [16]. Seki et al. reported that FB may cause severe constipation and urinary frequency [10]. A recent study by Yamane et al. [17] showed that PD patients with FB and KB frequently have deep venous thrombosis of their lower extremities. With LB, patient can develop lumbar pain, dyspnea, unsteadiness leading to falls, or lower leg numbness due to radiculopathy.

## Conclusion

Various postural abnormalities occur in patients with Parkinson’s disease, and are influenced by gender, age, disease severity, anti-Parkinson drugs, and other factors. However, these postural abnormalities do not occur independently, but are interrelated. Thus, from the viewpoint of the underlying pathophysiology, early detection and management, e.g., by physiotherapy, of one mild deformity could help to prevent or at least delay exacerbation of postural abnormalities.

### Ethics approval and consent to participate

All procedures performed in this study (involving human subjects) were in accord with the ethical standards of Tokai University Review Board and/or the National Research Committee and with the 1975 Declaration of Helsinki. All eligible individuals were informed of the purpose and methods of the study, and provided written informed consent.

### Consent for publication

Not applicable.

### Availability of data and materials

The dataset supporting the conclusions of this article is available upon request.

## Abbreviations

PD: Parkinson’s disease
H&Y stage: Hoehn & Yahr’s stage
NF: Neck flexion
FB: Fore-bent
KB: Knee-bent
LB: Lateral-bent
LED: Levodopa equivalent dose
DDA: Total dose of dopamine agonists
MSA: Multiple system atrophy
PSP: Progressive supranuclear palsy
MMSE: Mini-Mental State Examination
UPDRS: Unified Parkinson’s Disease Rating Scale
